# High-intensity activity is more strongly associated with metabolic health in children compared to sedentary time: a cross-sectional study of the I.Family cohort

**DOI:** 10.1186/s12966-021-01156-1

**Published:** 2021-07-06

**Authors:** Jonatan Fridolfsson, Christoph Buck, Monica Hunsberger, Joanna Baran, Fabio Lauria, Denes Molnar, Luis A. Moreno, Mats Börjesson, Lauren Lissner, Daniel Arvidsson, on behalf of the I.Family consortium

**Affiliations:** 1 Center for Health and Performance (CHP), Department of Food and Nutrition and Sport Science, Faculty of Education, University of Gothenburg, Box 300, SE-405 30 Gothenburg, Sweden; 2 Department of Biometry and Data Management, Leibniz Institute for Prevention Research and epidemiology – BIPS, Bremen, Germany; 3 School of Public Health and Community Medicine, Institute of Medicine, Sahlgrenska Academy, University of Gothenburg, Gothenburg, Sweden; 4 Institute of Health Sciences, Medical College, University of Rzeszów, Rzeszów, Poland; 5 Institute of Food Sciences, National Research Council, ISA-CNR, Avellino, Italy; 6 Department of Pediatrics, Medical School, University of Pécs, Pécs, Hungary; 7 GENUD (Growth, Exercise, Nutrition and Development) research group, Universidad de Zaragoza, Instituto Agroalimentario de Aragón (IA2), Instituto de Investigación Sanitaria de Aragón (IIS Aragón), Zaragoza, Spain; 8 Centro de Investigación Biomédica en Red de Fisiopatología de la Obesidad y Nutrición (CIBEROBN), Instituto de Salud Carlos III, Madrid, Spain; 9 Center for Health and Performance (CHP), Department of Molecular and Clinical Medicine, Institute of Medicine, Sahlgrenska Academy, University of Gothenburg, Gothenburg, Sweden; 10 Sahlgrenska University Hospital/Östra, Region of Western Sweden, Gothenburg, Sweden

**Keywords:** Obesity, Cardiovascular disease, Metabolic syndrome, Frequency filtering, Multivariate pattern analysis, Multicollinearity

## Abstract

**Background:**

Physical activity (PA) during childhood is important for preventing future metabolic syndrome (MetS). To examine the relationship between PA and MetS in more detail, accurate measures of PA are needed. Previous studies have only utilized a small part of the information available from accelerometer measured PA. This study investigated the association between measured PA and MetS in children with a new method for data processing and analyses that enable more detailed interpretation of PA intensity level.

**Methods:**

The association between PA pattern and risk factors related to MetS was investigated in a cross- sectional sample of children (*n* = 2592, mean age 10.9 years, 49.4% male) participating in the European multicenter I. Family study. The risk factors examined include body mass index, blood pressure, high-density lipoprotein cholesterol, insulin resistance and a combined risk factor score (MetS score). PA was measured by triaxial accelerometers and raw data was processed using the 10 Hz frequency extended method (FEM). The PA output was divided into an intensity spectrum and the association with MetS risk factors was analyzed by partial least squares regression.

**Results:**

PA patterns differed between the European countries investigated, with Swedish children being most active and Italian children least active. Moderate intensity physical activity was associated with lower insulin resistance (*R*^2^ = 2.8%), while vigorous intensity physical activity was associated with lower body mass index (*R*^2^ = 3.6%), MetS score (*R*^2^ = 3.1%) and higher high-density lipoprotein cholesterol (*R*^2^ = 2.3%). PA of all intensities was associated with lower systolic- and diastolic blood pressure, although the associations were weaker than for the other risk factors (*R*^2^ = 1.5% and *R*^2^ = 1.4%). However, the multivariate analysis implies that the entire PA pattern must be considered. The main difference in PA was observed between normal weight and overweight children.

**Conclusions:**

The present study suggests a greater importance of more PA corresponding to an intensity of at least brisk walking with inclusion of high-intense exercise, rather than a limited time spent sedentary, in the association to metabolic health in children. The methods of data processing and statistical analysis enabled accurate analysis and interpretation of the health benefits of high intensity PA that have not been shown previously.

**Supplementary Information:**

The online version contains supplementary material available at 10.1186/s12966-021-01156-1.

## Background

Physical activity (PA) promotes healthy development in children and has a major role in the prevention of cardiometabolic disease [1, 2]. Still, less than one third of European children and adolescents are sufficiently physically active [3]. Cardiometabolic disease mainly refers to obesity, hypertension and type-2 diabetes, which are lifestyle related risk factors for premature death [1, 4–6]. Though children are less likely to present with disease, exposure to unhealthy lifestyles, even in children, are associated with a greater risk for obesity as well as high blood pressure [7, 8]. These risk factors tend to cluster and in combination are often called metabolic syndrome (MetS) [9]. MetS is defined by central or total adiposity, hyperglycemia, dyslipidemia and elevated blood pressure and is typically quantified by combining standardized measures of these separate risk factors [9]. Similar to the risk factors alone, higher MetS score is associated with increased risk of cardiovascular disease and premature death [10]. Furthermore, higher PA level is associated with lower quantitative MetS scores [11]. However, more research is needed about the effect of high intensity PA [1].

To investigate the relationship between PA and risk factors for MetS, objective measurement of PA has become widespread in large-scale epidemiological research the last decade. Although the relationship between objectively measured PA and risk factors for MetS is stronger compared to self-reported PA [12], previous analyses have not taken advantage of all the information provided by objective methods. Objective measurement of PA is typically captured by an accelerometer worn by the participants for approximately 1 week [13]. Raw accelerometer data is collected at least 30 times every second [14], which is sufficient to capture PA with very high detail. However, reduction of PA information is common in the processing of raw accelerometer data before the output is used in further analyses. The most common processing method for raw accelerometer data is to generate ActiGraph counts [13]. The ActiGraph process involves a narrow frequency filter that removes most of the information related to high intensity PA captured by the accelerometer [15]. This is mainly caused by the step frequency during high intensity PA being 2.5–3 Hz whereas the low-pass half-power cut-off of the filter is at 1.66 Hz [15–17]. With increasing PA intensity, the step frequency increase, resulting in the ActiGraph filter removing a larger part of the information related to PA [16]. At high intensity PA only 80% of the information in the accelerometer signal remain [15]. After narrow frequency filtering, differentiation between moderate (MPA) and vigorous PA (VPA) is not possible [17]. The ActiGraph processing results in overestimation of time spent at high intensity PA since the filtered output interprets a substantial amount of MPA as VPA [18]. In children, this is an even more prominent issue because their step frequency is higher than adults and often children spend more time being active when compared to adults [16, 18]. By applying a wider frequency filter that is able to distinguish VPA from MPA, more accurate results are obtained [16, 19, 20].

Furthermore, processed accelerometer output represents PA intensity with a resolution of 1 to 60 s, referred to as epoch length. However, the PA intensity of these 1 to 60 s epochs is often heavily reduced to time distributed over three to five crude intensity levels of which the MVPA intensity is mainly considered [14]. Recent advances in research indicate dividing the PA output into substantially more (20+) intensity levels would allow researchers to take advantage of the detailed output retrieved from accelerometers [19, 21]. The detailed variables represent a spectrum of PA that can be used in further analyses to find patterns related to health outcomes such as MetS risk factors. The variables representing the PA spectrum are, however, highly collinear and analyses that can handle multicollinearity, such as Partial Least Squares Regression (PLS), must be applied [21].

The advancements in accelerometer data processing and statistical analysis have rarely been integrated into large national and international epidemiological research studies. Therefore, the aim of the current study was to investigate the association between PA and MetS in a sample of children from six European countries by taking advantage of the detailed information that accelerometer measured PA provides in combination with novel statistical approaches.

## Methods

### Study sample

The association between PA patterns and MetS was investigated using a cross-sectional subsample of the European multicenter I. Famliy study that extends the IDEFICS study [22]. The subsample consisted of children with available measurements of PA by triaxial accelerometer that participated in the I. Family study in 2013–2014 [22]. The main analytic sample included children from Italy, Hungary, Germany, Spain and Sweden. In addition, we used accelerometer data from a Polish research center following specific parts of the I. Family study protocol. Details of the study design and data collection have been previously published [22].

### Measurement of metabolic syndrome

The examination procedure have been described in detail previously [9, 23]. Blood glucose was measured by an enzymatic UV test, high-density lipoprotein cholesterol (HDL) was measured by a homogeneous enzymatic colorimetric test and triglyceride levels were measured by an enzymatic colorimetric test (Cobas c701, Roche Diagnostics GmbH, Mannheim, Germany). Serum Insulin levels were measured by luminescence immunoassay (AUTO-GA Immulite 2000, Siemens, Eschborn, Germany). Fasting levels of blood glucose and insulin were used to calculate insulin resistance (IR) by homeostatic model assessment [24].

All measures of MetS risk factors were standardized to age- and sex- specific z-scores with a mean of zero and standard deviation (SD) of one. Specifically, BMI was standardized using the definition from Cole and Lobstein [25]. The remaining risk factors, i.e. waist circumference, diastolic and systolic blood pressure, HDL, triglycerides and blood glucose, were standardized according to Ahrens et al. [9, 22]. Additionally, blood pressure was standardized with regard to height. Sex- and age-specific reference values were derived for children and adolescents using data collected in the IDEFICS and I. Family cohort [23]. Statistical modelling and handling of changes in measurement methods between IDEFICS and I. Family were described in detail as supplemental material in Börnhorst et al. [23]. Standardized waist circumference, hyperglycemia, blood pressure and dyslipidemia were combined into a MetS score as described previously [9]. The MetS score was calculated as the sum of the age- and sex standardized z-scores of waist circumference, IR, the mean of the z-scores of systolic (SBP) and diastolic (DBP) blood pressure and the mean of the z-scores of triglycerides and reversed HDL (multiplied by − 1). HDL was reversed because, contrary to the other variables, high values of HDL are associated with better health. In this way, waist circumference, hyperglycemia, blood pressure and dyslipidemia are weighted equally in the MetS score with a high number representing poor cardiometabolic health. In the Polish sample, only BMI data were available. Descriptive statistics of the MetS risk factors are displayed in Table 1.

**Table 1:** Descriptive characteristics and number of children with valid data from each study center

		All	Italy	Hungary	Germany	Spain	Sweden	Poland
Sex	% male	49.4%	52.0%	47.6%	47.2%	47.9%	48.8%	55.6%
	n	2592	588	567	436	497	299	205
Age (years)	mean	10.9	11.1	11.3	11.5	10.7	10.8	8.8
	SD	2.5	2.2	2.2	2.4	2.3	2.2	3.8
	n	2592	588	567	436	497	299	205
BMI (kg/m^2^)	mean	19.1	21.3	18.7	19.0	18.4	17.6	17.6
	SD	4.0	4.2	4.1	3.9	3.2	2.7	3.5
	n	2592	588	567	436	497	299	205
MetS score	mean	1.23	1.98	1.99	0.35	0.20	−0.33	
	SD	3.08	3.02	3.15	2.97	2.41	2.54	
	n	1183	335	369	208	141	130	0
SBP (mmHg)	mean	106.4	106.8	108.2	106.0	105.8	103.8	
	SD	9.2	9.2	10.3	9.1	8.3	7.5	
	n	2325	588	564	400	481	292	0
DBP (mmHg)	mean	64.7	64.5	65.8	63.1	65.0	64.3	
	SD	6.2	6.3	6.8	6.1	5.6	5.4	
	n	2325	588	564	400	481	292	0
HDL (mg/dl)	mean	59.5	56.8	56.3	60.0	65.8	59.3	
	SD	13.7	11.9	13.8	14.0	13.5	13.3	
	n	1691	432	439	267	398	155	0
IR (pg/ml∙mg/dl)	mean	1.88	1.74	2.62	1.77	1.10	1.17	
	SD	2.33	1.26	3.62	1.43	0.94	0.93	
	n	1228	340	388	212	151	137	0
SED (min/day)	mean	716.7	742.6	721.9	729.7	703.6	677.4	689.1
	SD	84.4	74.1	77.9	87.1	72.7	102.0	88.1
	n	2592	588	567	436	497	299	205
LPA (min/day)	mean	196.1	194.9	194.2	189.9	196.3	193.7	220.6
	SD	50.8	51.2	51.3	51.6	46.4	41.3	62.2
	n	2592	588	567	436	497	299	205
MPA (min/day)	mean	98.2	77.3	95.1	92.3	108.3	134.1	102.9
	SD	40.8	28.8	29.7	36.4	31.0	66.7	33.1
	n	2592	588	567	436	497	299	205
VPA (min/day)	mean	6.55	4.02	6.42	5.87	8.47	10.06	5.83
	SD	5.44	3.32	5.14	5.56	5.71	6.71	3.66
	n	2592	588	567	436	497	299	205
VVPA (min/day)	mean	2.46	1.17	2.34	2.19	3.35	4.72	1.58
	SD	3.14	1.64	2.69	2.56	3.03	5.42	1.75
	n	2592	588	567	436	497	299	205
MVPA (min/day)	mean	107.3	82.5	103.9	100.3	120.1	148.9	110.3
	SD	46.7	32.1	34.4	42.0	36.6	75.4	36.3
	n	2592	588	567	436	497	299	205

MetS metabolic syndrome score including waist circumference, blood pressure, blood lipids and insulin resistance. *BMI* body mass index, *SBP* systolic blood pressure, *DBP* diastolic blood pressure, *HDL* high density lipoprotein, *IR* insulin resistance from homeostatic model assessment, *SED* sedentary, *LPA* light physical activity (PA), *MPA* moderate PA, *VPA* vigorous PA, *VVPA* very-vigorous PA, *MVPA* moderate-to-vigorous PA

### Measurement of physical activity

Participants were instructed to wear a triaxial accelerometer (GT3x+, ActiGraph, Pensacola, FL, USA) over the right hip for 7 consecutive days, removing it during sleep and water-based activities. The accelerometers were set to record movement at a sample rate of 30 Hz and a range of ±6 *g*. Raw acceleration data was filtered using the 10 Hz frequency extended method (FEM) [18]. Filtered output from the three axes were combined to a single vector magnitude and reduced to three-second epochs. Because of varying compliance to removing the accelerometer during sleep, night-time between 23:00 and 06:00 was set to zero. Traditional cut-points were applied to the output representing time spent light PA (LPA), MPA, VPA and very vigorous PA (VVPA), which is equivalent to energy expenditure above 1.5, 3, 6 and 9 metabolic equivalents of task respectively [18]. Time spent below the first cut-point was considered sedentary (SED). Additionally, MPA, VPA and VVPA was summed into a variable representing MVPA. Non-wear time was defined as 60 min of consecutive zeros with allowance of up to 2 min of output up to the LPA cut-off [18, 26]. A valid day of measurement was defined as at least 10 hours of wear time and a valid measurement was defined as at least four valid days [14].

Traditional cut-points provide a very crude measure of PA patterns and remove much of the information available in the collected accelerometer data [21]. To enable a more in depth investigation of the detailed data that the accelerometers provide, the output was also divided into an intensity spectrum with 22 narrow intensity bins ranging from zero output to approximately 50% above the VVPA cut-off [19]. The number and width of bins were selected in order to cover the entire intensity range where movement occurs and enable comparison to previous literature [19]. The bin edges were 0, 40, 80, 160, 240 m*g*, increasing with 80 m*g* per variable until 1600 m*g* and above. Noteworthy is that 24.2 and 6.9% of the Pearson correlation coefficients between the different PA intensity spectrum variables were > 0.70 and > 0.90 respectively. For reference, 30.0 and 0% of the Pearson correlations between the traditional cut-points PA variables were > 0.70 and > 0.90 respectively. Traditional statistical analyses cannot handle multicollinearity to this extent [21]. Therefore, PLS analysis was applied, which is capable of handling this multicollinearity. Study characteristics and PA levels defined by the traditional cut-points are shown in Table 1.

### Statistical analyses

To visualize differences in PA patterns between study centers and between BMI categories [25], the PA intensity spectrum was standardized to z-scores representing each intensity variable. The country and weight group specific means of the standardized PA intensity spectrum was plotted as separate lines. Each mean was accompanied by a 95% confidence interval that was retrieved by bootstrapping 10^4^ times.

Participants’ characteristics, including age, sex, country, household income and parents’ education were considered as confounders that could affect the relationship between PA and MetS risk factors [27, 28]. Household income was a five-level categorical variable representing household income in relation to average country-specific household income. Parents’ education was a three-level categorical variable representing the highest education of the parents based on the International Standard Classification of Education (ISCED) 2011 [29]. In order to conduct a regression analysis, country, household income and parents’ education were treated as dummy variables.

The variables that compose the PA intensity spectrum are highly collinear and cannot be used in traditional statistical analyses [21], which was also the case in the present study as presented above. Therefore, a multivariate analysis approach by partial least squares regression (PLS) was used. PLS decomposes the predictor variables (independent) into multiple latent variables, referred to as PLS components, formed by maximizing the covariance with the response variable (dependent) [30]. Subsequently, the association between the PLS components and the response variable is analyzed. To investigate the association between PA and MetS, six separate PLS analyses were performed with the PA intensity spectrum as predictor variables and MetS score, BMI, SBP, DBP, IR and HDL as response variables in each respective model. To account for the effect of the confounding variables, four separate PLS models were generated for each of the six response variables. The three first models consisted of one model with the PA intensity spectrum as predictor variables, one model with the confounding variables as predictor variables and one model with both the PA intensity spectrum and the confounding variables as predictor variables. The forth model included the PA intensity spectrum as predictor variables but removed the influence of the confounding variables from the response variable before used in the PLS regression. This was done by using the residuals from a multiple linear regression with the confounding variables as independent variables and the initial response variable as dependent variable [11]. Traditional analysis of confounders divides the association to the response variable (dependent) that is shared, between PA variables and confounders to ensure the best model fit. This is not possible with multicollinear predictor variables. Therefore, the three first models above compare the strength of the association to the response variable between the PA variables and confounders separately and simultaneously. In this case, part of the association is shared between the PA variables and the confounders and net effect of PA cannot be isolated. In the fourth model, the association between the confounders and response is removed before input to the PLS model. Hence, all the association to the response variable that is shared between the PA variables and confounders is ascribed to the confounders exclusively. This represents the net association of the PA variables.

Predictor and response variables were standardized before input to the PLS models [30]. The number of PLS components in each model, referred to as model complexity, was based on cross-validation by Monte Carlo resampling with 1000 repetitions. A backwards selection procedure with a cut-off of half a standard deviation was used to ensure that the model complexity was significantly better than a model with fewer components [31]. The statistical significance of the PLS models was assessed by permutation tests with 10^4^ repetitions [32]. Significance level was set to *p* < 0.05. The strength of the PLS models was expressed as the cross-validated explained variance of the response variable (*R*^2^). Selectivity ratio plots were used to visualize the pattern of the relationship between the predictor and response variables [33]. The separate PLS components were combined to one vector by target projection. Subsequently, the selectivity ratio was calculated as the ratio of explained variance to total variance of the target-projected component followed by multiplication by the overall explained variance of the model [34]. This results in the predictor variables specific explained variance of the response variable, which was plotted as a spectrum with positive or negative values depending on the sign of the PLS coefficients. In summary, the selectivity ratio represents the PLS regression coefficients standardized to their predictive performance [33]. Confidence intervals of the selectivity ratio (95%) was calculated by bootstrapping with 10^4^ repetitions.

In the standardized mean plots and selectivity ratio plots, additional x-axes representing energy expenditure and locomotion speed are added as reference in order to facilitate understanding of PA intensity level [16, 18]. For the same purpose, the cut-points for the traditional PA intensity categories are indicated. Data processing and statistical analyses were performed in MATLAB 2020a (MathWorks, Natick, MA, USA). PLS analyses were performed using the MATLAB function ‘plsregress’ available in the Statistics and Machine Learning Toolbox.

## Results

**Fig. 1:**
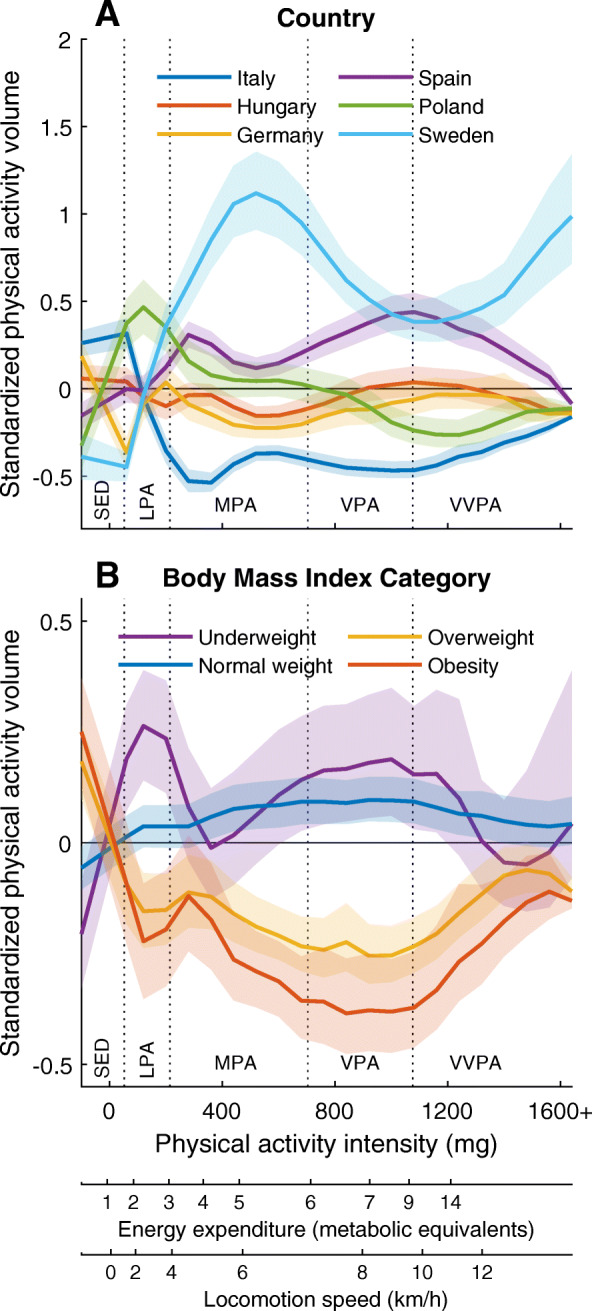
Standardized means (95% CI) of physical activity (PA) across the spectrum of PA intensity for different **A** countries and **B** BMI categories. Different scales for PA intensity are included as well as the cut-points for traditional PA intensity categories (dotted vertical lines) in order to facilitate understanding. SED sedentary time, LPA light PA, MPA moderate PA, VPA vigorous PA, VVPA very vigorous PA

PA patterns differed between the study centers located in six European countries. Specific numbers are presented in Table 1 and the pattern representing each country is visualized in Fig. 1A. Children from the Swedish study center spent least time SED and most time at the entire MVPA range. The children from the Spanish study center followed just behind the Swedish children, but spent more time than the children from the other participating countries across the MVPA range. In contrast, children from the Italian study center spent more time in SED and less time across the MVPA range compared to the other countries. Children representing the four BMI categories also displayed different PA patterns, visualized in Fig. 1B. Under- and normal weight children spent less time in SED and more time in PA, whereas overweight and obese children spent more time in SED and less time in PA.

Table 2 shows the strength and uncertainty of the PLS models. All PLS models, except for SBP and IR, were statistically significant according to the permutation tests. Among the MetS risk factors investigated, BMI showed the strongest association to the PA intensity spectrum in all models. Blood pressure, both SBP and DBP, showed the weakest associations.

**Table 2:** Strength of partial least squares regression models

	PA and confounders	PA only	Confounders only	PA vs residuals
BMI	18.3%^**^	3.4%^**^	11.1%^**^	6.5%^**^
MetS	12.5%^**^	2.7%^**^	11.0%^**^	4.2%^**^
SBP	1.8%^**^	1.2%^**^	4.3%^**^	0.1%
DBP	1.8%^**^	1.4%^**^	4.5%^**^	0.4%^*^
HDL	7.9%^**^	1.3%^**^	7.5%^**^	1.1%^**^
IR	10.8%^**^	1.7%^**^	10.5%^**^	0.5%

Confounders include child’s age, child’s sex, household income, parents’ education. Residuals after removing the association to confounders. ^*^ indicates *p* < 0.05 and ^**^ indicates *p* < 0.01 from permutation tests. MetS metabolic syndrome score including waist circumference, blood pressure, blood lipids and insulin resistance. *BMI* body mass index, *SBP* systolic blood pressure, *DBP* diastolic blood pressure, *HDL* high-density lipoprotein, *IR* insulin resistance

**Fig. 2:**
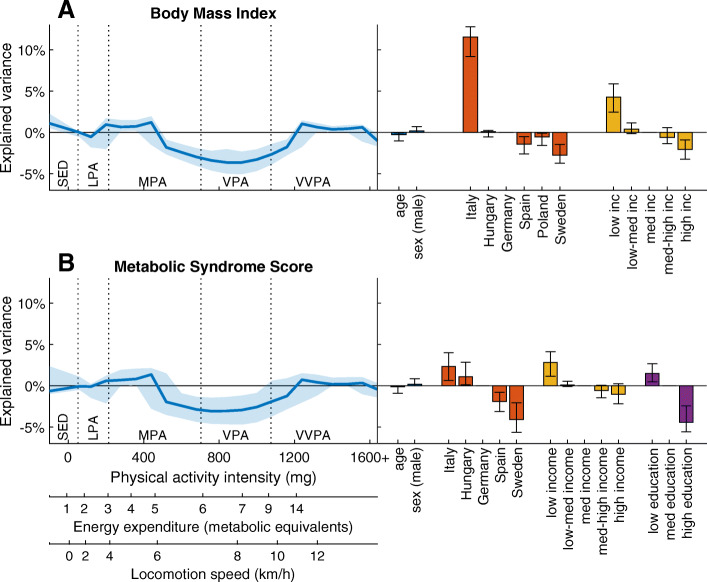
Selectivity ratio plots showing the explained variance (95% CI) and direction (positive values mean positive associations) of the predictor variables in the PLS models with A) BMI and B) MetS as response variables. The left panel displays the explained variances of the physical activity intensity spectrum (main predictor). The right panel shows the explained variances by age, country, income and education (confounders). Different scales for PA intensity (x-axes) are included as well as the cut-points for traditional PA intensity categories (dotted vertical lines) in order to facilitate understanding. SED sedentary time, LPA light PA, MPA moderate PA, VPA vigorous PA, VVPA very vigorous PA

The association between PA together with the confounders and BMI and MetS score is visualized by selectivity ratio plots in Fig. 2. More PA from the mid MPA range to above the VVPA cut-off was associated with lower BMI whereas time spent SED was associated with higher BMI. Survey center differences are also observed; the Italian children had BMI scores that were greater than the Spanish and Swedish participants. Higher household income was associated with lower BMI. The magnitude of the association to the other confounders were within the same range as the PA pattern. Like BMI, PA within the mid MPA range to above the VVPA cut-off was associated with a lower MetS score. Similarly, high household income and parents’ education was associated with a lower MetS score. Further, the association differed by country with Swedish children having the lowest MetS score. The highest explained variance in the PA intensity spectrum (peak) was 3.6 and 3.1% with BMI and MetS score, respectively.

**Fig. 3:**
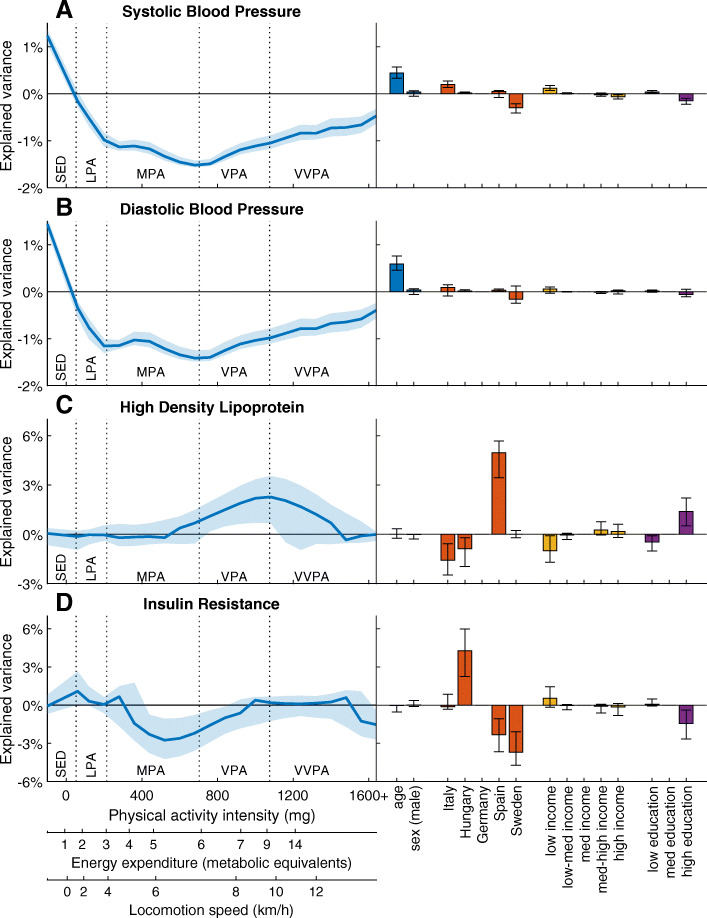
Selectivity ratio plots showing the explained variance (95% CI) and direction (positive values mean positive associations) of the predictor variables in the PLS models with A) SBP, B) DBP, C) HDL and D) IR as response variables. The left panel displays the explained variances of the physical activity intensity spectrum (main predictor). The right panel shows the explained variances by age, country, income and education (confounders). Different scales for PA intensity (x-axes) are included as well as the cut-points for traditional PA intensity categories (dotted vertical lines) in order to facilitate understanding. SED sedentary time, LPA light PA, MPA moderate PA, VPA vigorous PA, VVPA very vigorous PA

Figure 3 shows the associations of PA and confounders with SBP, DBP, HDL and IR. Less SED and more PA at all intensities were associated with lower SBP and DBP. In addition, age was associated with higher SBP and DBP. A positive association between PA and HDL was found in the VPA and VVPA ranges while there was a negative association between MPA and VPA to IR. In HDL and IR, the association differed more by country, which was not the case in SBP and DBP. The highest explained variance in the PA intensity spectrum (peak) in the SBP, DBP, HDL and IR models were 1.5, 1.4, 2.3 and 2.8% respectively. The PA patterns of the association to the residuals of the risk factors after removing the association to the confounders were similar to the models that included the confounders as predictor variables. These patterns are available as supplementary material 1.

## Discussion

The present study adds novel information to the existing knowledge regarding geographical differences of PA-patterns among children in six European countries, as well as information of the specific importance of high intensity PA to prevent MetS in children. The main associations between PA and decreased risk of MetS are apparent in VPA consistently. In addition, the results suggest that high-end MPA is required for health benefits in children, whereas lower end MPA was not associated with metabolic health in most cases. The association between SED and MetS risk factors was generally weak. Furthermore, the associated PA patterns differed between MetS risk factors. The detailed investigation of PA pattern without the limitations of traditional cut-points was made possible by the novel methods of data processing and statistical analysis. Specifically, the PLS models showed the overall strength and uncertainty of the relationship between PA pattern and MetS risk factors. These PA patterns were graphically visualized as an intensity spectrum to facilitate interpretation. In addition, income and education were associated with PA levels and MetS risk, therefore, considering this in the association is a strength.

Firstly, in the current study, geographical PA differences were shown in higher detail across the entire PA intensity spectrum. The overall geographical PA differences between study centers were similar to previous studies of European children [3]. In addition, the actual levels of MVPA were higher in the current study. This is explained mainly by the use of a shorter epoch length, 3 s compared to 60 s in older studies [35]. A 60-s epoch length implies that bursts of PA shorter than 60 s might not be counted as MVPA although the activity intensity might have been high enough. Most of the geographical differences represent a proportional shift of time between SED and MVPA where e.g. Swedish children had less SED and more MVPA, Italian children more SED and less MVPA and most other countries a distribution somewhere in between. Another exception in the country specific PA patterns is the Swedish children’s VVPA level. The high end of VVPA is equivalent to very short bursts of high intensity PA that cannot be performed for a prolonged time. Therefore, this PA is likely not related to any significant increase in total energy expenditure, but rather represents different behaviors that may be important for health and development. It could for example be related to sports participation that is higher in Sweden compared to the other countries [36]. The specific PA pattern in the Swedish children but also in the Spanish children may be related to their specific pattern of MetS risk factors discussed below.

Secondly, the current results suggest that the higher part of MPA but not the lower part is associated with lower BMI and MetS score, and that the overall PA patterns associated with BMI and MetS score were highly similar. The PA intensities that were associated with these outcomes ranges from the mid MPA range to the lower part of VVPA, corresponding to brisk walking to fast running. Similarities between the patterns of these risk factors are expected since excess adiposity is included in the calculation of the MetS score. Still, the almost identical patterns suggest that BMI could be sufficient for estimating metabolic health, instead of more invasive measures including blood samples included in the MetS score. In children, BMI may be an early sign of future MetS, and therefore highly interesting. The association between higher level of MVPA and lower BMI and MetS score is consistent with previous literature [1]. However, the results of the present study demonstrate the benefit of considering PA as an intensity spectrum instead of crude cut-points based variables. Since the lower half of the intensity range representing MPA is not associated with lower BMI, the association would likely be attenuated and possibly missed in traditional cut-points based analysis. Furthermore, the standardized means figure suggests that the main BMI related difference in PA pattern is between normal and overweight children. This suggests that the relationship between PA and BMI is non-linear with the strongest association in the range between normal- and overweight children.

An interesting finding was the difference between HDL and IR in their association pattern with PA. While the association for IR started from the mid MPA, the association for HDL started at higher PA intensities. Previous results from intervention studies in adults suggest that MPA is not sufficiently intense to increase HDL, but that VPA is required [37]. Our data in children are congruent with these results. Furthermore, previous research in youths suggests that high volume MPA could be more beneficial than shorter bursts of VPA for improving IR [38]. In our study, children spent on average 15 times more time at MPA compared to VPA. Since VPA is defined as twice as high energy expenditure as MPA [18], MPA accounts for 7.5 times as much energy expenditure as VPA. This suggests that IR could be more related to total energy expenditure, whereas HDL could be more related to high intensity exercise. The importance of high intensity exercise for HDL may be further supported by the finding that the Spanish children had higher VPA-VVPA and HDL. These findings may imply that children are required to include VPA in their daily activities to maintain a lower MetS risk. Further, a composite MetS score might not be an optimal outcome to investigate the association between PA and metabolic health since PA levels have different associations with individual risk factors. Similarly, the components of the MetS score are weighted equally, although the association with metabolic health may not be the same for all components. The association between the components and metabolic health is more complex than the MetS score indicates and a high MetS score does not necessarily imply that individuals display clinical symptoms of diabetes or cardiovascular disease.

SBP and DBP showed unique patterns compared to the other risk factors with significant associations across the PA intensity spectrum, although these models were weak. In addition, these patterns were the only ones where a relationship with SED time was found. Although recent recommendations on PA include limiting children’s SED time, the association between SED and metabolic health is weaker compared to PA and metabolic health [1, 2]. Furthermore, the association to all risk factors diminishes at the highest end of the VVPA spectrum. This is probably due to too little time accumulated at this intensity level, rather than a true effect [19]. Altogether, rather than limiting sedentary time, our data suggest a greater importance of more PA corresponding to an intensity of at least brisk walking with the inclusion of high-intensity exercise is more important for metabolic health in children.

The effect of the confounders used in the PLS models varied. In the BMI, MetS score, HDL and IR models, the effect of country roughly represented the differences found in Table 1. As expected, higher income and education per se were associated with better health in these models [39]. Although the response variables were standardized for age and sex, these variables were included as confounders as they are related to PA [27, 28]. Sex was not a significant predictor in any of the models. Age was only significant in the SBP and DBP models, the same models where country, income and education displayed weaker associations compared to the other models. Although all risk factors were standardized with regard to age and sex, this was done in a much larger sample and the variation in the investigated sub-sample that included PA measurement might not reflect the overall sample. Furthermore, the strength of the SBP and DBP models including PA and confounders were weaker than the models only including the confounders. In addition, the models of the residuals suggests that there were little to no net association between PA and blood pressure. On the other hand, the strength of the model with PA and confounders was higher than the sum of the PA and confounders models alone regarding BMI. In this case, the combined effect of PA and confounders generated latent variables with a considerably stronger association with BMI than the PA and confounders alone. Similarly, the net association between PA and BMI from the residuals model was stronger than the PA only model.

The peak explained variation in the PA spectrum was consistently higher than the overall explained variation in the PA only models, although the differences between risk factors were proportional. This suggests that taking the variation related to the confounders into consideration strengthens the PA patterns association to MetS. In all risk factors, the strength of the PA variable only model was much weaker than the model with only confounders. Therefore, the influence of these variables on children’s health was greater than the PA levels. However, the selectivity ratio plots suggest that the influence of PA was roughly the same as income and education, whereas geographical differences seem to be of greater importance.

In traditional multiple linear regression, the independent variables have a unique association to the dependent variable. Inserting confounders in addition to e.g. variables representing PA as independent variables can be interpreted as removing the effect of these confounders from the association between PA and the dependent variable. Hence, a specific part of the association can only be ascribed to one independent variable. However, since the PLS model decomposes the predictor variables into fewer latent variables, a large proportion of the explained variation shown in the selectivity ratio plots is shared between multiple response variables. This means that the association with the response variable is shared between predictor variables and the effect of the confounders cannot be interpreted in the same way as in traditional multiple linear regression. Including confounders as predictor variables in the PLS analysis is a novelty of the current study since it has not been applied in multivariate analysis of PA previously. This gives a detailed insight into the PA and confounders shared association to the MetS risk factors. In previous studies, confounders have been handled by removing their association to the response variable and using the residuals in the PLS model [11, 40]. This ascribes all the shared association to the confounders exclusively and has been included in the present study to assess the independent association of PA. Both methods have limitations regarding the interpretation in relation to traditional multiple linear regression, but can together be used to interpret the results in a similar way. In summary, the models using residuals yields the same results as the models including confounders in the model with regard to the associations to different risk factors and the PA patterns of the associations.

The strength of the associations in the PLS models cannot be directly compared to studies using traditional multiple linear regression since PLS removes parts of the information in the predictor variables to handle collinearity. A recently published study by Aadland et al. is to our knowledge, the only other study where PLS analysis is used to investigate the association between PA and MetS in an international sample of children [40]. This study used residuals from a multiple linear regression to handle confounders. The strengths of the models with comparable risk factors in this previous study were 4.2% for MetS, 4.2% for waist circumference to height ratio, 2.7% for IR and 1.7% for total cholesterol to HDL ratio. The corresponding model strength in the present study with PA and the risk factor residuals were slightly higher with regard to MetS, roughly the same regarding BMI and lower for IR and HDL. The definition of MetS used differed between studies and waist circumference to height ratio and total cholesterol to HDL ratio was used in the previous study compared to BMI and HDL in the present study. In a separate study by Aadland et al., a national sample of children was investigated using PLS [11]. This study found stronger associations between PA and the MetS risk factors investigated. This could be explained by a more homogenous study sample.

Compared to the present study, Aadland et al. used older methods for capturing and processing PA data [40]. Only vertical movement was considered, an epoch length of 60 s was used and the raw data was processed with the very narrow ActiGraph filter. The use of solely vertical acceleration may limit capturing of running at higher intensities [41] and other activities such as playing soccer and cycling [42]. A longer epoch length is not able to capture children’s intermittent PA pattern with short bursts of running and jumping followed by less intense activity [35]. Applying the narrow ActiGraph filter in the processing of accelerometer data instead of the much wider filter in the FEM used in the current study, filters out a majority of the accelerometer signal related to high intensity PA [16]. This implies that the association between PA output and intensity level is very weak in the MVPA range and different PA intensities cannot be separated [18]. Consequently, time spent at MPA is mixed up with VPA and VVPA causing a majority of the PA being overestimated and the effect of different intensities being undistinguishable [18, 19]. These limitations together obstruct interpretation of the effect of different intensity levels within the MVPA range. Further, in the study by Aadland et al. the PA intensity spectrum analyzed ranges from 0 to 8000 counts per minute. The upper limit is equivalent to running at 10 km/h [43] or 9 metabolic equivalents [44] which corresponds to the VVPA cut-point. Hence, in the current study the PA pattern investigated includes intensities approximately 50% higher than in the study by Aadland et al. and the PA processing method allows an accurate interpretation of high intensity PA.

Although the results of the current study suggest that higher levels of PA are associated with lower MetS risk, the cross-sectional study design limits inference about causation. The results regarding the importance of different PA intensity levels should be interpreted as what intensities are the most influential in the cross-sectional association between PA and metabolic health. Longitudinal observational studies suggest that increased body weight causes decreased PA rather than the opposite [45, 46]. However, there is strong evidence that intervention studies increasing PA leads to favorable results regarding metabolic disease risk factors [1]. In addition, PA seems to be associated with IR independent of BMI [47]. Furthermore, in children with low cardiorespiratory fitness, PA may be more beneficial [48].

## Conclusions

This study found the strongest association between PA and decreased metabolic disease risk at vigorous intensity. Although PA above moderate intensity was beneficial for metabolic health, the association patterns were not consistent between MetS risk factors. In contrast, the association between SED and metabolic disease was weak for most risk factors. Furthermore, geographical differences in PA patterns were demonstrated. The results suggest a greater importance of more PA corresponding to an intensity of at least brisk walking with inclusion of high-intense exercise, rather than a limited time spent sedentary, in the association to metabolic health in children. The detailed analysis of PA pattern was enabled by accurate processing of accelerometer data, investigation of the intensity spectrum with high resolution and application of multivariate statistics.

## Supplementary Information

**Additional file 1.**

## Data Availability

Ethical restrictions prohibit the authors from making the minimal data set publicly available because this study is based on highly sensitive data collected in young children. However, interested researchers can contact the IDEFICS and I. Family consortium (http://www.ideficsstudy.eu/Idefics/ and http://www.ifamilystudy.eu/) to discuss possibilities for data access.

## Abbreviations

BMI: Body mass index
DBP: Diastolic blood pressure
FEM: Frequency extended method
HDL: High-density lipoprotein
IR: Insulin resistance
LPA: Light physical activity
MetS: Metabolic syndrome
MPA: Moderate physical activity
PA: Physical activity
SBP: Systolic blood pressure
SD: Standard deviation
SED: Sedentary
VPA: Vigorous physical activity
VVPA: Very-vigorous physical activity
