# Supplementary material for: High-intensity activity is more strongly associated with metabolic health in children compared to sedentary time: a cross-sectional study of the I.Family cohort
Source: Int J Behav Nutr Phys Act. 2021 Jul 6;18:90. doi: 10.1186/s12966-021-01156-1 (PMC8261968; doi:10.1186/s12966-021-01156-1)
Supplement: Supplementary file 1 — Additional file 1. [file 12966_2021_1156_MOESM1_ESM.docx]

**Supplementary material 1.** Selectivity ratio plots showing the explained variance (95% CI) and direction (positive values mean positive associations) of the PA intensity spectrum in the PLS models with residuals of A) BMI, B) MetS, C) diastolic blood pressure and D) high density lipoprotein as response variables. Residuals were retrieved from a multiple linear regression with age, country, income and education as independent variables (confounders). Different scales for PA intensity (x-axes) are included as well as the cut-points for traditional PA intensity categories (dotted vertical lines) in order to facilitate understanding. SED sedentary time, LPA light PA, MPA moderate PA, VPA vigorous PA, VVPA very vigorous PA.
